# Identification of a novel immune checkpoint regulator and potential therapeutic antibody target in oncology

**DOI:** 10.1186/2051-1426-3-S2-P195

**Published:** 2015-11-04

**Authors:** Nathan Sallee, Artur Karasyov, David Bellovin, Ryan Liang, Jacqueline de la Torre, Servando Palencia, Ernestine Lee, Kevin Hestir, Thomas Brennan, Luis Borges, Arthur Brace, Brian Wong

**Affiliations:** 1 Five Prime Therapeutics, Inc., South San Francisco, CA, USA

## 

Antibody blockade of immune checkpoint regulators such as PD-1 and CTLA4 has been shown to be an effective cancer treatment strategy; however, a large percentage of patients still do not respond to existing therapies. Discovery of additional immune checkpoints and development of antibody therapeutics against them are likely critical to address this unmet patient need. We generated a comprehensive library of essentially all human extracellular proteins and screened proteins in this library *in vitro* and *in vivo* for the ability to modulate immune responses or tumor growth. As a result of these screens, we identified a number of novel immune checkpoints^1^.

One such protein, referred to herein as Novel Checkpoint 1, was originally identified through its inhibitory activity on anti-CD3-stimulated human T cell proliferation. To confirm its activity as an immune checkpoint, we demonstrated that the native protein expressed on an antigen-presenting cell line could inhibit antigen-stimulated CD8+ T cell activation. Furthermore, blocking antibodies against this protein relieved the inhibition. This inhibitory activity translated to a murine system, as the mouse ortholog and blocking antibodies behaved similarly in murine T cell activation assays. Overexpression of the protein in mouse syngeneic tumor models resulted in increased tumor growth, consistent with inhibition of anti-tumor immune responses. Novel Checkpoint 1 is expressed primarily on activated and regulatory T cells in humans and mice – an expression profile similar to those of PD-1 and CTLA4. Additionally, it is expressed on 40-70% of tumor-infiltrating T cells while only on 10-15% of circulating T cells from those tumor-bearing mice. We are currently evaluating the anti-tumor activity of blocking antibodies in mouse tumor models, either alone or in combination with other checkpoint blocking antibodies. Taken together, we believe that these data demonstrate that this newly discovered protein may act as a checkpoint regulator in tumors and that blocking antibodies against it have potential as a novel cancer immunotherapeutic.
